# Homocysteine and Its Relationship to Asymptomatic Carotid Stenosis in a Chinese Community Population

**DOI:** 10.1038/srep37361

**Published:** 2016-11-21

**Authors:** Jiaokun Jia, Anxin Wang, Jing Wang, Jianwei Wu, Xiujuan Yan, Yong Zhou, Shengyun Chen, Xingquan Zhao

**Affiliations:** 1 Department of Neurology, Beijing Tiantan Hospital, Capital Medical University, Beijing, China; 2 China National Clinical Research Center for Neurological Diseases, Beijing, China; 3 Center of Stroke, Beijing Institute for Brain Disorders, Beijing, China; 4 Beijing Key Laboratory of Translational Medicine for Cerebrovascular Disease, Beijing, China; 5 Department of Epidemiology and Health Statistics, School of Public Health, Capital Medical University, Beijing, China; 6 Beijing Institute of Heart, Lung and Blood Vessel Disease, Beijing Anzhen Hospital, Capital Medical University, Beijing, China

## Abstract

Little is known about the association between homocysteine (Hcy) and asymptomatic CAS in the healthy population. The purpose of this study was to investigate the relationship between Hcy levels and asymptomatic CAS in a Chinese community population. The current study included 5393 participants who were age of 40 years or older, and free of stroke, transient ischemic attack, and coronary artery disease. Demographic and clinical variables were investigated, and the presence of CAS was assessed by Color Doppler Ultrasound. A multivariate logistic regression was used to examine the association between Hcy levels and asymptomatic CAS. 361 (6.69%) participants were diagnosed with asymptomatic CAS, who had higher Hcy levels compared with those without (p-value for trend = 0.0001). After adjusting other possible risk factors, Hcy > 19.3μmol/L was considered as an independent indicator of asymptomatic CAS (OR 1.53, 95%CI 1.05–2.23; p-value for trend = 0.0265), but with a difference between participants with diabetes and without [OR (95%CI): 2.89(1.02–8.22) vs. 1.42(0.95–2.12); P interaction < 0.05]. In this large-population, community-based study, Hcy is an independent indicator of asymptomatic CAS, especially in patients with diabetes.

Stroke remains a major global health problem with a significant increase in stroke burden in the world over the last two decades^1^. The Asymptomatic Carotid Stenosis and Risk of Stroke (ACSRS) Study Group found the rate of ipsilateral strokes in patients with moderate asymptomatic carotid stenosis (CAS) was 5.3%. Also, the risk of ipsilateral stroke was higher in patients with progressive asymptomatic CAS^2^ compared with those without.

Numerous researches have already confirmed that the total homocysteine(Hcy) level is a risk factor of ischemic stroke (IS), especially in younger subjects and those with concomitant hypertension^3^,^4^. Also, a prospective study of a large stroke population with a median follow-up period of 48 months found elevated Hcy levels in the acute phase of ischemic stroke can predict mortality, especially in stroke patients with the large-vessel atherosclerosis subtype^5^. Although mild elevations in Hcy levels have modest effects on cardiovascular risk, high Hcy levels promotes atherothrombosis^6^. And proposed mechanisms of the complex changes within the blood vessel wall produced by Hcy^7^ included oxidative stress, proinflammatory effects, impaired endothelium-mediated platelet inhibition and suppressive growth of endothelial cells and vascular smooth muscle cells^7^,^8^,^9^. Most studies focus on the relationship between Hcy and restenosis after carotid endarterectomy(CEA)^10^ or symptomatic CAS^2^,^4^, while only few focuses on the relationship between Hcy and asymptomatic CAS^11^,^12^.

We hypothesized that Hcy levels may represent a potentially modifiable risk factor of asymptomatic CAS. The objective of this cross-sectional study was to assess the correlation between Hcy level and the prevalence of asymptomatic CAS in a Chinese healthy population.

## Results

### Baseline characteristics

The baseline characteristics according to the classification of asymptomatic CAS were shown in Table 1. A total of 5393 participants were enrolled in the study. Compared with the group of subjects without asymptomatic CAS, the group of subjects with asymptomatic CAS 1) had more elders, males and current smokers (P < 0.05); 2) was more likely to have hypertension, diabetes mellitus and the use of antihypertensive and antidiabetic drugs (P < 0.05); 3) had higher value of some biomarkers, such as hs-CRP and Hcy (P < 0.01); and 4) had higher Hcy levels (p-value for trend = 0.0001). Whereas there was no significant difference in hyperlipidemia, atrial fibrillation between the two groups.

### The correlations between the levels of serum homocysteine and asymptomatic carotid stenosis

**Figure 1:**
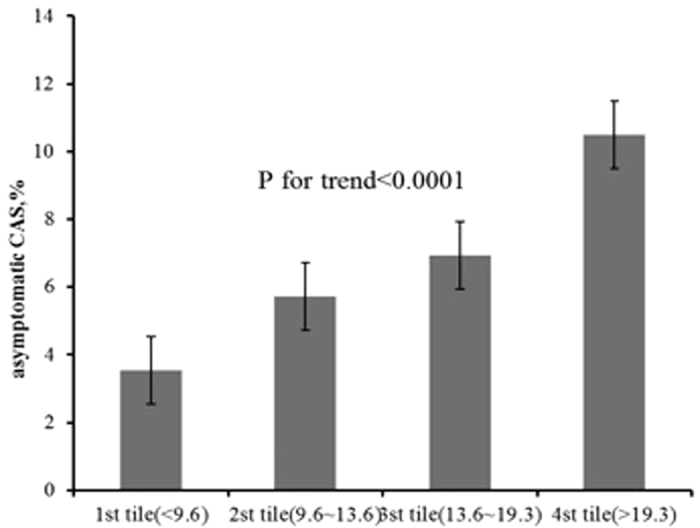
Prevalence of asymptomatic carotid stenosis(CAS) stratified by quartile of homocysteine.

Prevalence of asymptomatic CAS stratified by quartile of Hcy could be found in Fig. 1, which showed that the Hcy level was positively associated with the prevalence of asymptomatic CAS (p-value for trend < 0.001).

Prior to adjusting for any possible confounders, Hcy levels ≥9.6μmol/L were associated with the presence of asymptomatic CAS (Hcy 9.6–13.6μmol/L, OR 1.66, 95%CI, 1.14–2.40; Hcy 13.6–19.3μmol/L, OR 2.03, 95%CI, 1.42–2.91; Hcy >19.3 μmol/L, OR 3.15, 95%CI, 2.25–4.42; p-value for trend <0.001). After adjusting for age, gender, BMI, hypertension, diabetes, hyperlipidaemia, current smoking status and hs-CRP, only Hcy >19.3μmol/L remained as an indicator of asymptomatic CAS (multivariate-adjusted OR 1.53, 95%CI 1.05–2.23; p-value for trend = 0.0265). (Table 2)

Further analysis of the interaction effects of age, gender, hypertension, diabetes and hyperlipidaemia on the association between Hcy levels and the prevalence of asymptomatic CAS showed that there was a significant difference between diabetic and non-diabetic subjects (P for interaction 0.04). In diabetic subjects, those of Hcy >19.3 μmol/L were more likely to suffer asymptomatic CAS(OR 2.89, 95%CI 1.02–8.22), however, this tendency was not found in non-diabetic subjects(OR 1.42, 95%CI 0.95–2.12). Other possible interaction factors have no significant effect on the association (all of them P > 0.05), although some subgroups’ OR were significant(P < 0.05). (Table 3)

## Discussion

In this large sample of 5393 participants from Kailuan study cohort, a Chinese community population cohort, association of Hcy level and asymptomatic CAS was observed, and that the prevalence of asymptomatic CAS had elevated trends with higher quartile of Hcy. Also, the study suggested that elevated Hcy level was an independent indicator for the presence of asymptomatic CAS, especially when Hcy level reached 19.3μmol/L and above. Although numerous clinical studies have demonstrated that Hcy was an independent risk factor for cardiovascular disease, stroke and symptomatic CAS^4^,^13^,^14^, and even was associated with poor outcome of the patients with ischemic stroke^5^,^15^,^16^, few study discussed the relation between Hcy and asymptomatic CAS. A cross-sectional study of 1041 subjects from the Framingham Heart Study^11^ has showed that elevated Hcy was associated with an increased risk of CAS, however, the subjects of their study aged from 67 to 96, which were older than the subjects enrolled in our study. Also, the subjects of some other studies were primarily non-Asian, while subjects of our study were all Chinese^12^. Meanwhile, Catena *et al*. suggested that elevated plasma Hcy levels were associated with carotid artery plaques and intima-media thickness (IMT) in hypertensive patients^17^. Kim *et al*. found that Hcy was independently associated with asymptomatic CAS in patients undergoing coronary artery bypass grafting^18^. As CAS always occurred together with hypertension and coronary stenosis^19^, the conclusions of many studies may not be able to apply to all healthy subjects with asymptomatic CAS. Based on the Kailuan study, subjects in our study were randomly selected, all of which were without history of stroke, transient ischemic attack, and coronary disease at baseline. Therefore, we further certified that elevated Hcy level was associated with asymptomatic CAS in a large Chinese healthy community population.

The multiethnic population-based Northern Manhattan Study(NOMAS)^12^ showed Hcy was independently associated with carotid plaque morphology and increased plaque area. Similarly, which has also been found from a cross-sectional study in Chinese adults derived from a reference population of the Kailuan Cohort Study^20^. Carotid plaque formation, together with CAS, were the results of atherosclerosis which might be caused by hyperhomocysteinemia. But the mechanism is still controversial. Hyperhomocysteinemia could produce complex changes within the blood vessel wall^7^. The proposed mechanisms include oxidative stress, proinflammatory effects such as expression of tumor necrosis factor-α and inducible nitric oxide (NO) synthase (iNOS), impaired endothelium-mediated platelet inhibition and suppressive growth of endothelial cells and vascular smooth muscle cells^7^,^8^,^9^. In addition, genetic abnormalities and nutritional deficiencies of B vitamins could result in hyperhomocysteinemia and then lead to atherosclerosis^6^,^21^,^22^.As atherosclerosis is the main cause of CAS, our study suggested that high level of Hcy was an independent risk factor of asymptomatic CAS.

Moreover, the analysis for the interaction effects showed that diabetes had an interaction effect on the association between Hcy levels and asymptomatic CAS. Although many studies^23^,^24^,^25^ found that the level of homocysteine was higher in diabetics than those without in different genders, different races, and in patients with coronary artery disease, no study suggest it in participants with asymptomatic CAS. Also, some studies found that individuals with Type 1 and Type 2 diabetes are more susceptible to the effects of homocysteine than non-diabetic subjects^26^ and homocysteine might be associated with microvascular complication of retinopathy^23^,^27^,^28^ and nephropathy^29^ in diabetes. Therefore the interaction of DM with high levels Hcy on the risk of CVD may have an influence on the management of primary and secondary prevention in DM patients^30^, even in those already with asymptomatic CAS. Meanwhile, The most likely reason of the interaction effect was that insulin resistance or insulin secretion insufficient could influence Hcy level, and there was a negative relationship between insulin and Hcy level^31^,^32^. Meanwhile, diabetes and insulin resistance may also cause endothelial injury as well as atherosclerosis through systemic oxidative stress^33^,^34^. In addition to insulin resistance, studies have shown that some inflammatory cytokines, such as C-reactive protein (CRP), sialic acid, tumor necrosis factor-α (TNF-α) and interleukin-6 (IL-6), associated with diabetes, can also cause atherosclerosis^35^,^36^.

To the best of our knowledge, the study is one of the first studies to assess the relationship between Hcy levels and the prevalence of asymptomatic CAS in a large Chinese community-based study. However, the potential limitations also merit consideration. Firstly, a cross-sectional study as ours cannot prove a causal relationship which usually has to be shown in a longitudinal investigation. The results of our study only allow concluding on an association between Hcy and asymptomatic CAS, while the role of Hcy as a risk factor for asymptomatic CAS may be shown in follow-up investigations. Secondly, all the participants were from area and same ethnic group. This would limit the application of the findings to a population with broader geographic and ethnic diversity. Thirdly, although ultrasonic method has been regarded as a noninvasive and convenient screening method to diagnose CAS, it is not as accurate as DSA for determining CAS. In addition, the plasma concentrations and intakes of vitamin B and folate were not collected and adjusted in our study, which may have mild effects on our results.

In summary, subjects with higher Hcy levels had a mildly increased prevalence of asymptomatic CAS in a large-population, community-based study, and Hcy is an independent indicator of asymptomatic CAS, especially in patients with diabetes.

## Methods

### Study population

From June 2010 to June 2011, the baseline survey of Asymptomatic Polyvascular Abnormalities Community study(APAC), employing a proportion of the large population of the Kailuan study which included a total of 101,510 employees and retirees (81,110 men) of the Kailuan (Group) Co. Ltd, a large coal mine industry located in Tangshan, Hebei Province^37^, has been completed. This study is a population-based, prospective, long-term follow-up observational study and has two phases- cross-sectional phase and longitudinal phase. The inclusion criteria were as follows: (1) age of 40 years old and older; (2) no history of stroke or transient ischemic attack; (3) no history of coronary disease. In the end, a total of 5440 participants were eligible and recruited in APAC. Standard protocol was described previously^38^. In the first cross-sectional phase, all study participants have undergone extensive clinical, laboratory and bilateral carotid duplex ultrasound examinations. However, fasting total plasma Hcy hasn’t been examined in 47 participants. Therefore, we enrolled 5393 participants in the study.

### Assessment of homocysteine(Hcy)

Blood samples were drawn by trained phlebotomists from the subjects after overnight fasting. The venous blood samples in tubes containing trisodium ethylenediaminetetraacetic acid were immediately placed on ice after antecubital venipuncture. Blood samples were then centrifuged for 10 minutes at 3000 rotations per minute at 25 °C. After separation, plasma samples were used within 4 hours. Fasting plasma glucose was measured using the hexokinase/glucose-6-phosphate dehydrogenase method. Total cholesterol and triglyceride were measured enzymatically according to the manufacturer’s instruction (interassay coefficient of variation <10%; Mind Bioengineering Co. Ltd., Shanghai, China). All biochemical variables were measured using an autoanalyzer (Hitachi 747; Hitachi, Tokyo, Japan) at the central laboratory of the Kailuan General Hospital. The Hcy levels were stratified in accordance to the quartile categories of serum Hcy concentration- Q1 (plasma total Hcy levels <9.6 μmol/L), Q2 (9.6 μmol/L ≤ plasma total Hcy levels <13.6 μmol/L), Q3 (13.6 μmol/L ≤ plasma total Hcy levels <19.3 μmol/L) and Q4 (plasma total Hcy levels >19.3 μmol/L).

### Assessment of carotid stenosis(CAS)

Each participant underwent a bilateral carotid duplex ultrasound examination (Philips iU-22 ultrasound system, Philips Medical Systems, Bothell, WA) to evaluate CAS as a part of their standard diagnostic workup. Bilateral carotid arteries, including the common carotid arteries (CCA), internal carotid arteries (ICA) and vertebral arteries (VA), were all examined with the participants in a supine position, head turning to the contralateral side. Both sides of carotid arteries were extensively evaluated. The carotid ultrasound examination results were then reviewed by two independent operators. Discrepancies between their evaluations were resolved by consensus. CAS diagnosis was made according to the peak systolic flow velocity (PSV) criteria that was published^39^. Briefly to say, the PSV for different arteries were: >125 cm/s for the CCA and ICA; >170 cm/s for the VA. In addition, when visible plaque and lumen narrowing were seen, it would also be taken into consideration for CAS diagnosis regardless of PSV. CAS was diagnosed when at least one of the studied arteries showed evidence of stenosis.

### Assessment of demographic variables and cardiovascular risk factors

Information on demographic variables, including age, ethnic group (Han), gender, marital status (married), current smoking status and alcohol consumption, was collected via questionnaires which were conducted in person by trained research doctors. The participants were classified into two groups according to whether they have asymptomatic CAS or not.

Information on cardiovascular risk factors mainly contained hypertension, diabetes mellitus, hyperlipidemia, atrial fibrillation, body mass index (BMI) level and high-sensitivity C-reactive protein (hs-CRP). Blood pressure was measured on the left arm to the nearest 2 mmHg using a mercury sphygmomanometer with a cuff of the appropriate size. Hypertension was defined as the presence of a history of hypertension, or a systolic blood pressure ≥140 mmHg/a diastolic pressure ≥ 90 mmHg, or the consumption of antihypertensive medication. Diabetes mellitus was diagnosed if the subject was currently undergoing treatment with insulin or oral hypoglycemic agents, or the fasting blood glucose level was ≥126 mg/dl, or the subject has a past history of diabetes mellitus. Hyperlipidemia was defined as the presence of a history of hyperlipidemia, or the total cholesterol level ≥220 mg/dl/, triglyceride ≥150 mg/dl, or the consumption of medication. Atrial fibrillation (AF) was diagnosed using electrocardiography. All participants had a standard unfiltered 12-lead ECG. ECG criteria used for the detection of AF included 1) the absence of P waves; 2) the presence of coarse fibrillatory waves; and 3) the presence of irregular QRS complex. Meeting all the above mentioned 3 criteria, the participants would be diagnosed AF by ECG. Body mass index (BMI) was calculated as body weight (kg) divided by the square of height (m^2^) and was categorized according to the classification system established by the National Institutes of Health (<25, 25 to 30, and >30).Blood samples were collected at the antecubital vein in the morning after an overnight fasting. For all participants, hs-CRP levels were assessed.

### Quality control (QC)

Within a few weeks of the initial baseline survey in a particular community, QC survey was done, involving around 1% of the participants randomly selected from that community with repeat questionnaires and measures on selected items. During the course of the survey, regular central monitoring was also undertaken to assess the distribution of certain key variables, the time delay with blood processing and the consistency of data collected, both overall and by individual staff. On-site monitoring visits were also undertaken every 6 months by staff from Kailuan general hospital.

### Statistical analyses

Statistics were performed with the SAS software, version 9.3 (SAS Institute Inc., Cary, NC, USA). We described continuous variables by their median (interquartile range) and categorical variables were described as percentages. We used the Student’s t-test for non-paired samples for the comparison of normally distributed parameters and the Wilcoxon test for the comparison of non-parametric variables. The Chi squared test was applied for the comparison of categorical variables. The trend of the prevalence of asymptomatic CAS with increasing Hcy level was tested by the Chi-square trend test. Multivariate logistic regression analyses were to calculate odds ratios (OR) and 95% confidence intervals (CI) for the associations of Hcy level with asymptomatic CAS. Model 1 adjusted for age and gender; Model 2 adjusted for age, gender, BMI, hypertension, diabetes, hyperlipidemia, antihypertensive drugs, lipid-lowering drugs, antidiabetic drugs, current smoking and hs-CRP. Finally, for each model, a trend test was performed after the Hcy level was entered into the model and treated as a continuous variable. Additionally, diabetes and other potential indicators were also evaluated to assess if there was any significant interaction between these variables and the relationship between Hcy levels and asymptomatic CAS presence. Two-sided P-values were reported for all analyses. A P-value < 0.05 was considered to be statistically significant.

### Ethical approval

The study was approved by both the Ethics Committee of the Kailuan General Hospital and Beijing Tiantan Hospital, in compliance with the Declaration of Helsinki. Written informed consent was obtained from all participants. All the experiments described were performed in accordance with the approved guidelines.

## Additional Information

**How to cite this article**: Jia, J. *et al*. Homocysteine and Its Relationship to Asymptomatic Carotid Stenosis in a Chinese Community Population. *Sci. Rep.* **6**, 37361; doi: 10.1038/srep37361 (2016).

**Publisher’s note:** Springer Nature remains neutral with regard to jurisdictional claims in published maps and institutional affiliations.

## Figures and Tables

**Table 1:** Baseline demographic characteristics and cardiovascular risk factors between groups with/without asymptomatic carotid stenosis.

	Without Asymptomatic CAS (n = 5032)	With Asymptomatic CAS (n = 361)	*p*
**Age, y, Median(Q1-Q3)(n)**	52.32(45.64–60.90)	57.02(46.20–74.05)	<0.0001
**Ethnic group (Han) n(%)**	4962 (98.6%)	354(98.1%)	0.3575
Gender n(%)			<0.0001
Male	2924(58.1%)	302(83.7%)	
Female	2108(41.9%)	59(16.3%)	
**Current smoking n(%)**	1548(30.76%)	169(47.0%)	<0.001
**Hypertension n(%)**	2395(47.6%)	196(54.3%)	0.0142
**Diabetes mellitus n(%)**	589 (11.7%)	58(16.1%)	0.0185
**Hyperlipidemia n(%)**	2330(46.3%)	156(43.2%)	0.2744
**Antihypertensive drugs n(%)**	957(19.0%)	90(24.9%)	0.0061
**Lipid-lowering drugs n(%)**	69(1.4%)	6(1.7%)	0.6485
**Antidiabetic drugs n(%)**	293(5.8%)	32(8.9%)	0.0190
**Atrial fibrillation n(%)**	69(1.4%)	9/361 (2.5%)	0.1038
**BMI level(kg/m**^**2**^**) Median(Q1-Q3)(n)**	24.77(22.76–27.05)	24.09(21.97–26.57)	<0.0001
<25 n(%)	2656 (52.8%)	228 (63.2%)	0.0003
25–30 n(%)	2041 (40.6%)	120 (33.2%)	
>30 n(%)	335 (6.7%)	13 (3.6%)	
**hs-CRP,mg/dL Median(Q1-Q3)(n)**	1.0(0.5–2.14)	1.1(0.6–2.95)	0.0054
**Hcy (μmol/L) Median(Q1-Q3)(n)**	13.4(9.5–19.0)	16.3(11.9–22.8)	<0.0001
1st tile(<9.6) n(%)	1316 (26.2%)	50 (12.9%)	<0.0001
2st tile(9.6~13.6) n(%)	1257 (25.0%)	76 (21.1%)	
3st tile(13.6~19.3) n(%)	1256 (25.0%)	92 (25.6%)	
4st tile(>19.3) n(%)	1203 (23.9%)	143 (39.6%)	

Values are median (interquartile range) or number (percent).CAS: carotid stenosis; BMI: body mass index; hs-CRP: high-sensitivity C reactive protein; Hcy: homocysteine.

**Table 2:** Multivariate-adjusted OR and 95% CI for asymptomatic carotid stenosis.

	Quartile of homocysteine (μmol/L)				p-value for trend
	1st tile(<9.6)	2st tile(9.6 ~ 13.6)	3st tile(13.6 ~ 19.3)	4st tile(>19.3)	
**Unadjusted**					<0.001
OR(95%CI)	1.00 (Reference)	1.66(1.14–2.40)	2.03(1.42–2.91)	3.15(2.25–4.42)	
p		0.008	<0.001	<0.001	
**Model 1**					0.0216
OR(95%CI)	1.00 (Reference)	1.22(0.83–1.78)	1.20(0.82–1.74)	1.52(1.05–2.19)	
p		0.32	0.35	0.03	
**Model 2**					0.0265
OR(95%CI)	1.00 (Reference)	1.23(0.84–1.81)	1.24(0.85–1.81)	1.53(1.05–2.23)	
p			0.28	0.27	0.03

OR: odd ratio; CI: confidence interval.

Model 1: adjusted for age and gender.

Model 2: adjusted for age, gender, BMI, hypertension, diabetes, hyperlipidemia, antihypertensive drugs, lipid-lowering drugs, antidiabetic drugs, current smoking and hs-CRP.

**Table 3:** Multivariate-adjusted OR and 95%CI for asymptomatic carotid stenosis according to quartile of homocysteine, stratified by age, gender and selected risk factors.

	Quartile of homocysteine (μmol/L)				Continuous Scale	P interaction
	1st tile(<9.6)	2st tile(9.6 ~ 13.6)	3st tile(13.6 ~ 19.3)	4st tile(>19.3)		
Age						0.76
<60y	1	1.33(0.85–2.10)	1.56(0.99–2.46)	1.60(1.01–2.53)*	1.01(0.99–1.02)	
≥60y	1	0.98(0.46–2.06)	0.85(0.41–1.76)	1.03(0.51–2.10)	0.99(0.97–1.01)	
gender						0.44
Female	1	1.27(0.68–2.39)	0.72(0.30–1.70)	1.71(0.76–3.87)	1.02(0.98–1.06)	
Male	1	1.26(0.77–2.04)	1.39(0.88–2.22)	1.60(1.02–2.52)*	1.00(0.99–1.01)	
Hypertension						0.74
No	1	1.53(0.91–2.57)	1.36(0.79–2.33)	2.05(1.22–3.44)*	1.01(0.99–1.03)	
Yes	1	0.97(0.55–1.70)	1.08(0.63–1.85)	1.11(0.65–1.91)	1.00(0.98–1.01)	
Diabetes						0.04
No	1	1.13(0.74–1.71)	1.30(0.87–1.95)	1.42(0.95–2.12)	1.00(0.99–1.01)	
Yes	1	2.19(0.77–6.27)	0.90(0.28–2.86)	2.89(1.02–8.22)*	1.02(0.99–1.05)	
Hyperlipidemia						0.59
No	1	1.63(0.97–2.76)	1.59(0.94–2.68)	2.16(1.29–3.64)*	1.01(0.99–1.02)	
Yes	1	0.88(0.50–1.53)	0.90(0.52–1.56)	1.01(0.59–1.74)	1.00(0.98–1.02)	

*P < 0.05.

OR: odd ratio; CI: confidence interval.

Adjusted for age, gender, BMI, hypertension, diabetes, hyperlipidemia, antihypertensive drugs, lipid-lowering drugs, antidiabetic drugs, current smoking and hs-CRP.
